# Analysis of molecular mechanism of Chinese medicine Jinhong decoction (JHD) in synergistically treating sepsis and COVID-19 based on network pharmacology

**DOI:** 10.1371/journal.pone.0339457

**Published:** 2025-12-18

**Authors:** Jinghan Fan, Yang Yang, Fan Zhang, Xiaowei Wu, Jiajun Li, Li Liang, Qi Tang, Bo Yan, Jiancheng Zhang, Xuming Pan, Guangjun Jin, Changlong Wei, Pingping Han, Yuzhou He

**Affiliations:** 1 The Second Affiliated Hospital of Zhejiang Chinese Medical University (Xinhua Hospital of Zhejiang Province), Hangzhou, China; 2 Zhejiang Chinese Medical University, Hangzhou, China; Hazara University, PAKISTAN

## Abstract

Sepsis and COVID-19 are the two mutually-reinforcing risk factors, whose interaction drastically increases mortality rate. Jinhong decoction (JHD) as a Chinese medicine exhibits clinical efficacy against them, but related action mechanism remains to be explored. To this end, using network pharmacology, this study screened active ingredients of JHD and their targets from TCMSP, HERB, PubChem and SwissTargetPrediction databases as well as the targets for these two diseases from DisGeNET, OMIM, Drugbank, TTD, and GeneCards. By intersecting drug and disease targets, we identified common targets and constructed a drug-ingredient-target network. GO and KEGG enrichment analyses revealed key target-related signaling pathways, and transcriptomics analysis further validated tissue distribution of these targets and their expressions. Our identified six key target genes (*AKT1*, *MMP9*, *ICAM1*, *TLR4*, *BCL2*, and *HIF1A*) were mainly involved in the regulations of immunometabolism, inflammation, and cell survival in both diseases. Functional enrichment analysis indicated that JHD displayed synergistic efficacy against both diseases by simultaneously modulating HIF-1, TNF, and NF-κB signaling pathways. Tissue distribution analyses of these 6 key target genes revealed that CD33 + myeloid cells, fetal lung cells, and bronchial epithelial cells might play an important role in treating both diseases. Overall, this study demonstrates that JHD treats sepsis and COVID-19 through a multi-ingredient, multi-target, and multi-pathway inter-related mechanism, exhibiting a great application potential.

## Introduction

Sepsis and COVID-19 are two pandemic diseases with increasing global incidence and substantially similar pathophysiological characteristics [1], exhibiting shared symptoms including systemic hyperinflammation, immunological dysregulation, and multi-organ failure. Their clinical treatments are challenged by high mortality rate and intricate complications [2]. The “homotherapy for heteropathy” theory in traditional Chinese medicine (TCM) highlights that different diseases with homogeneous pathogenesis could be treated with the same therapy. This therapy offers novel pathways for developing broad-spectrum anti-inflammatory medicines. Jinhong decoction (JHD), as a classic traditional Chinese medicine soup, is composed of rhubarb, red vine, and dandelion, exhibiting the efficacy of clearing heat and removing toxins, activating blood circulation, and clearing up the bowels. The previous study has demonstrated that JHD significantly attenuates hyperinflammatory symptoms, accelerates febrile resolution in co-diagnosed sepsis-COVID-19 cases, and reduces the critically ill patients’ acute physiology and chronic health evaluation II (APACHE II) score and sequential organ failure assessment (SOFA) score [3,4]. In a clinical trial, the treatment with JHD significantly increased the number of lymphocytes in sepsis-COVID-19 patients, which mechanistically confirmed its anti-inflammatory and immunoregulatory dual potencies. Although therapeutic efficacy of JHD against sepsis-COVID-19 comorbidity has been demonstrated in numerous clinical trials, its action mechanism has not been systematically elucidated.

In recent years, network pharmacology has emerged as a discipline integrating multi-omics data and molecular interaction networks, providing a framework for analyzing the ingredient-target-pathway polypharmacological synergism in TCM formula [5,6]. With the latest breakthrough in systematic biology and artificial intelligence, the traditional Chinese medicine has been successfully combined with computational network pharmacology, thus providing a holistic perspective for understanding traditional medicine and a new research paradigm for analyzing complicated disease pathegenesis and exploring multi-target intervention strategies of Chinese medicine [7]. Previous studies have identified principal bioactive ingredients (such as rhubarb anthraquinones and red vine lignans) in JHD, and these ingredients can modulate sepsis progression by inhibiting the NF-κB/MAPK inflammatory pathway and regulating apoptosis and autophagy [8,9]. Moreover, patients with severe COVID-19 exhibit pathological phenotypes such as over-activation of the TLR4/NF-κB pathway, vascular endothelial damage, and lymphocyte depletion [10]. The above findings jointly suggest that JHD may have a potential to achieve cross-disease therapy by modulating common targets. Although mono efficacy of JHD against sepsis and COVID-19 alone has been clinically validated [4,11], whether JHD has dual efficacy against both sepsis and COVID-19 in a patient suffering from both diseases, and the related molecular mechanisms remains largely unknown.

Considering this, the present study screened the active ingredients and related gene targets contained in JHD using network pharmacology analysis method, and then identified the common target genes of these two diseases and active signaling pathways as well as tissue/cell distribution of these target genes, and differentially expressed genes. Our analysis revealed that despite their distinct etiologies (causative agent infection for sepsis [12,13] versus virus infection for COVID-19 [14]), both diseases exhibited the common molecular mechanisms by which hyperactive pro-inflammatory pathways induced an abnormal immune response [15]. Our results showed that JHD regulated the expression of key target genes in sepsis and COVID-19 in specific tissues, thereby restoring immune homeostasis and influencing disease prognosis. Our multi-level analyses revealed that JHD exhibited a synergistic therapeutic efficacy against both diseases through multiple ingredients, targets, and pathways, adopting a mechanism known as “homotherapy for heteropathy” framework. This study reveals for the first time the molecular mechanism by which JHD achieves synergistic treatment for both sepsis and COVID-19, providing valuable reference for subsequent experimental validation and clinical application of JHD.

## Materials and methods

### Screening of active ingredients and drug target prediction of JHD

JHD was composed of 3 Chinese medicines, namely, rhubarb, red vine, and dandelion. The active ingredients of JHD were screened based on the Traditional Chinese Medicine Systems Pharmacology Database and Analysis Platform (TCMSP, https://www.old.tcmsp-e.com), and the key words related to the above three traditional Chinese medicines were retrieved to obtain all the chemical ingredient information of JHD. According to the screening thresholds of oral bioavailability (OB) ≥ 30% and drug-likeness (DL) ≥ 0.18, the key active ingredients of Chinese medicines rhubarb and red vine were obtained [16]. The OB and DL thresholds were set, as previously reported in TCM network pharmacology. Specifically, an OB ≥ 30% is typically required for favorable *in vivo* absorption and systemic exposure of oral compounds [17,18], while a DL ≥ 0.18, as proposed by the TCMSP database, serves as an empirical cutoff for drug-like properties [19]. These thresholds ensured the preservation of natural bioactive constituents of JHD, meanwhile achieving a balance between JHD pharmacokinetic properties and medicine features. Since the TCMSP database contained no information of Chinese medicine dandelion, its chemical ingredients were obtained from the HERB database (http://herb.ac.cn/), and the screening thresholds included MW ≤ 500, p ≤ 5, H-bond donor ≤ 5, and H-bond receptor ≤ 5 [20]. The eligible active ingredients were inputted into the PubChem (https://pubchem.ncbi.nlm.nih.gov/) database to retrieve and download their molecular structure formulae and PubChem CIDs, and the corresponding simplified molecular input line entry system (SMILES) formulae were retrieved [21]. The SMILES formulae were imported into the SwissTargetPrediction (http://swisstargetprediction.ch/) platform, and then the corresponding target genes of the active ingredients whose species was “Homo sapiens” were screened according to the chemical similarity between the active ingredient ligands. All targets screened through SwissTargetPrediction were included, with repeated data removed. These target genes were named after target names in the UniProt database [22]. A list of all abbreviations is provided in S1 Table.

### Screening for disease targets of sepsis and COVID-19

The targets of sepsis and COVID-19 were also screened by searching the key words “sepsis” and “COVID-19” from the 5 disease databases including the Gene-Disease Association Database (DisGeNET: https://www.disgenet.org), the Therapeutic Target Database (TTD: https://db.idrblab.net/ttd/), the Drug Target Database (DRUGBANK: https://go.drugbank.com/), the Human Gene Database (GeneCards: https://www.genecards.org), and the Comprehensive Human Mendelian Genetics Database (OMIM: https://www.omim.org). The target genes from the above 5 disease databases were combined with the repeated disease target genes eliminated to obtain final disease target set of sepsis and COVID-19 [23].

### Construction of protein interaction network (PPI) and screening of core targets

The active ingredient targets and disease targets were intersected to obtain the shared targets of JHD and diseases. According to these intersection targets, the Venn diagram was plotted by using the Jvenn platform (https://bioinfogp.cnb.csic.es/tools/venny/). The intersection targets of JHD and sepsis-COVID-19 were uploaded to the STRING database (https://www.string-db.org/, version 11.0) to construct the protein-protein interaction (PPI) network. Subsequently, the key targets were screened from this network with score threshold set as > 0.40, species set as Homo sapiens, other default settings retained, final export file as tsv file [24]. In the PPI network, the edges represent protein-protein connection, and the more the edges, the stronger the connection. Then we imported the tsv files into Cytoscape (version 3.10.3) and used Network Analyzer for topological analysis to screen core targets [25]. The degree, mediator value, and median degree of each node were calculated to further identify the key targets for sepsis and COVID-19 treatment with JHD.

### Gene Ontology (GO) functional enrichment analysis and Kyoto Encyclopedia of Genes and Genomes (KEGG) pathway enrichment analysis

The intersection targets were imported into the DAVID database for GO and KEGG enrichment analysis to identify the biological pathways and reveal mechanisms related to sepsis and COVID-19 treatment with JHD [26]. The GO enrichment analysis involved three categories, namely, biological process (BP), molecular function (MF), and cellular component (CC). With species set as “Homo sapiens”, biological signaling pathways were screened based on *P*-value, and *P* < 0.05 were considered statistically significant. Then the top twenty GO terms ranked by P-value in each of BP, CC, and MF categories from the GO functional enrichment analysis were inputted into the Microbiome Bioinformatics website (https://www.bioinformatics.com.cn/) to generate bubble plots for visualization analysis. After KEGG enrichment analysis, the top KEGG pathways significantly enriched with intersection target genes were selected, followed by literature-based verification of their research significance, to draw the bubble plots for visualization analysis. Finally, the ingredients, targets, pathways, drugs, and diseases obtained from the above steps were imported into Cytoscape software to plot the drug-ingredient-target-disease-pathway map.

### Network construction of drug active ingredients and potential therapeutic targets

The active ingredients of JHD and potential therapeutic targets (drug-disease intersection targets) were imported into Cytoscape software to construct a “drug-active-ingredient-potential-therapeutic-target” network.

### Tissue distribution of therapeutic targets in key pathways

The key targets in the screened signaling pathways were imported into Cytoscape software for network topology analysis. Node proteins were ranked primarily by connectivity (degree). The key targets with degree values > the median degree were screened and imported into the BioGPS platform (http://biogps.org). Tissue information with correlation coefficient > 0.9 was retrieved with the database set as “GeneAtlas U133A, gcrma” and other default settings retained [27,28]. The top 5 tissues or cells with highest gene expressions of each gene were presented in a bar graph.

### Differential expression analysis of core target genes

We obtained gene expression profiles of sepsis and COVID-19 patients from the Gene Expression Omnibus (GEO) (https://www.ncbi.nlm.nih.gov) (including GSE95233 gene set and GSE171110 gene set). GSE95233 gene set included 51 sepsis patient samples and 22 healthy control samples with an age range of 25–85 years (GPL570 platform) [29,30] (S2 Table). GSE171110 gene set contained 44 severe COVID-19 patient samples and 10 healthy control samples with an age range of 29–74 years (GPL16791 platform) [31] (S2 Table). Using limma package (version 3.58.1) in R software (version 4.3.3), the differentially expressed genes (DEGs) in comparison of sepsis vs. healthy samples were identified from the GSE95233 dataset, with the thresholds of adjusted *p*-value < 0.05 and |log_2_FC| > 0.5. Using DESeq2 package (version 1.42.1), DEGs in comparison of COVID-19 vs. healthy samples were identified from the GSE171110 dataset, with the thresholds of adjusted *p*-value < 0.05 and |log_2_FC| > 0.4. The ggplot2 package (version 3.5.1) was used to quantify the expressions of core targets in the control and disease groups (*p* < 0.05) and to draw the volcano plot of DEGs.

## Results

### A total of 189 active compounds and 611 predicted drug targets are screened from 3 Chinese herbal medicine components in JHD

A total of 189 active compounds were obtained from TCMSP and HERB online databases, of which 92 active compounds were from rhubarb, 25 from red vine, and 72 from dandelion in JHD. In terms of OB ≥ 30% and DL ≥ 0.18 criteria, these 189 active compounds were further screened, and finally 13 effective active ingredients were obtained from rhubarb, 4 from red vine, and 27 from dandelion.

Subsequently, the targets of the aforementioned active ingredients were predicted based on the PubChem database on the Swiss Target Prediction platform with species set as “Homo sapiens”. After eliminating repeated targets, ultimately a total of 611 drug targets of 3 Chinese herbal medicines in JHD were identified.

### A total of 54 drug-disease targets are obtained by intersecting disease targets (sepsis and COVID-19) with drug targets of JHD

**Fig 1:**
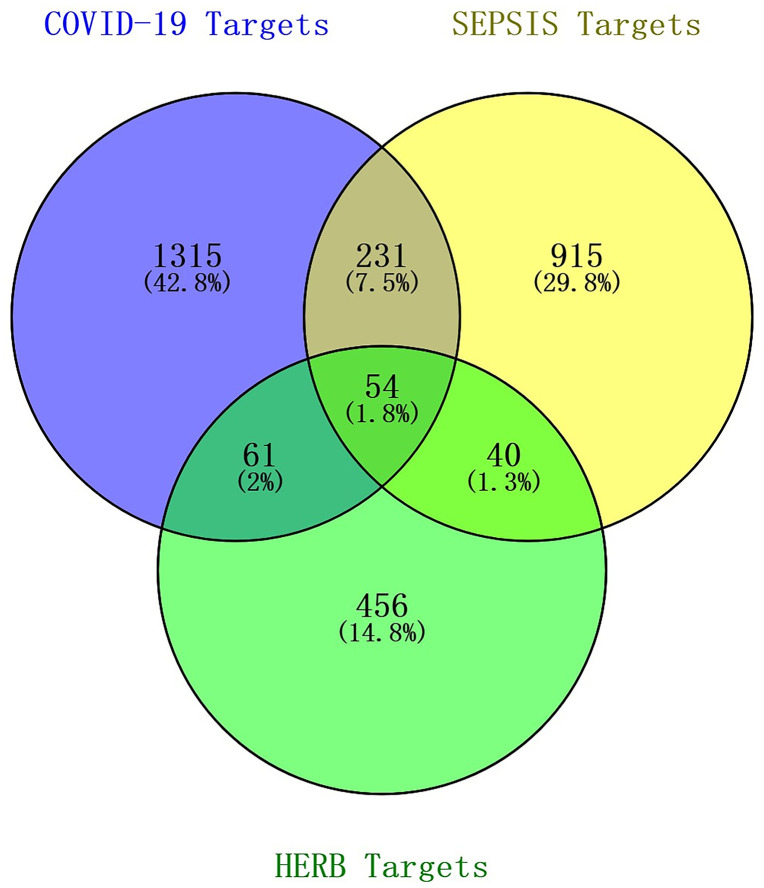
Identification of the drug-target interactions. Venn diagram of drug-disease intersection targets (drug, JHD; disease, sepsis and COVID-19).

Known target genes related to sepsis and COVID-19 were screened from 5 databases including the DISGNET database, TTD database, DRUGBANK database, GeneCards database and OMIM database. Then we combined the target genes from each database, removed repeated disease targets, and finally obtained 4,271 sepsis disease targets and 13,885 COVID-19 disease targets. The 611 drug targets of 3 Chinese herbal medicine components in JHD and 18,156 disease targets (sepsis and COVID-19) were intersected to obtain 54 drug-disease intersection targets. These intersection target data were imported into Jvenn platform to create a Venn diagram (Fig 1).

### Six core therapeutic targets for sepsis and COVID-19 treatment with JHD were identified through protein-protein interaction network (PPI) and topological analysis

The above-mentioned 54 intersection targets were uploaded to the STRING database to obtain the PPI network (S1 Fig). There were 54 nodes and 626 edges in the PPI network. The average node degree value in the network was 23.2, and the average central clustering coefficient was 0.733.

**Fig 2:**
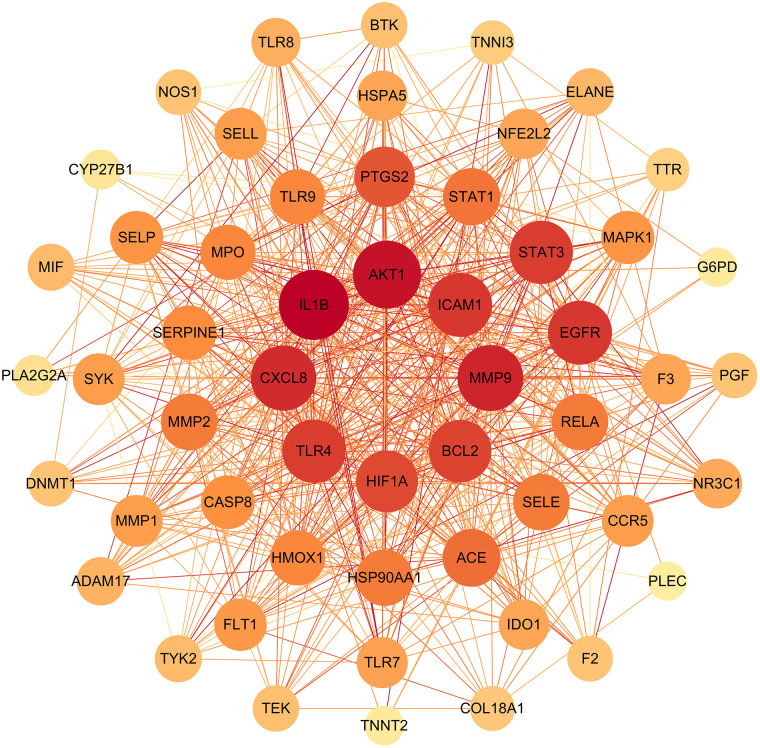
Protein–protein interaction (PPI) network by Cytoscape. Protein-protein interaction (PPI) network diagram of 54 intersection (drug-disease) targets.

The PPI network was constructed by using Cytoscape, and topological analysis was performed using Network Analyzer. Proteins were ranked primarily based on connectivity (degree value). Nodes represented individual protein-coding genes, and the connecting lines (edges) indicated protein-protein interaction relationships. Node size and node color depth were positively correlated with degree value and node importance, and the color depth of the connecting lines (edges) was positively correlated with the degree of association between nodes. The top 6 core therapeutic targets were screened (degree value > 35), including *AKT1* (degree = 46), *MMP9* (degree = 43), *ICAM1* (degree = 40), *TLR4* (degree = 39), *BCL2* (degree = 38), and *HIF1A* (degree = 37) (Fig 2).

### Ten key pathways for sepsis and COVID-19 treatment with JHD are identified through GO and KEGG pathway enrichment analyses

The DAVID database (http://david.ncifcrf.gov) was used to conduct GO functional enrichment analysis of the identified 54 intersection targets to further explore 3 functional categories including biological process (BP), molecular function (MF), and cellular component (CC) and to identify signaling pathways involved in the treatments of sepsis and COVID-19 by JHD. In GO enrichment analysis, a total of 441 GO terms were significantly enriched, of which 310 GO terms were annotated to BP category, mainly including positive regulation of interleukin-8 production, response to lipopolysaccharide, inflammatory response, positive regulation of angiogenesis, and positive regulation of interleukin-6 production; 86 GO terms were annotated to MF category, majorly including identical protein binding, heparin binding, endopeptidase activity, peptidase activity, and protease binding; and 45 GO terms were annotated to CC category, primarily including extracellular space, cell surface, external side of plasma membrane, protein-containing complex, and plasma membrane.

KEGG pathway analysis identified a total of 111 enriched regulatory pathways. Afterwards, 10 pathways were selected from these 111 significantly enriched pathways according to both P-value ranking and research significance supported by literature, which were presented in Table 1 and S3 Table.[Subxref6]

**Table 1:** Ten significantly enriched signaling pathways involved in sepsis and COVID-19 treatment with JHD based on KEGG pathway enrichment analysis.

KEGG signaling pathway	*P*-value
AGE-RAGE signaling pathway in diabetic complications	1.43 × 10^-14^
Lipid and atherosclerosis	5.93 × 10^-14^
HIF-1 signaling pathway	1.13 × 10^-12^
Pathways in cancer	8.08 × 10^-11^
Fluid shear stress and atherosclerosis	4.79 × 10^−10^
TNF signaling pathway	2.82 × 10^−8^
PD-L1 expression and PD-1 checkpoint pathway in cancer	5.02 × 10^−8^
Influenza A	5.83 × 10^−8^
Tuberculosis	9.43 × 10^−8^
NF-kappa B signaling pathway	1.69 × 10^−7^

**Fig 3:**
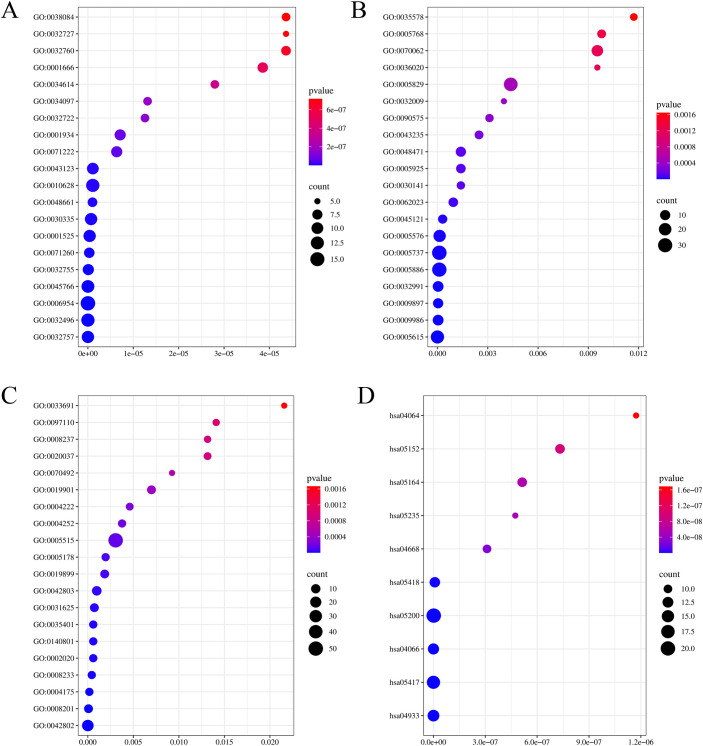
GO and KEGG enrichment analysis. **(A-C)** Top 20 GO terms annotated to 3 functional categories by GO enrichment analysis **(A)** BP, biological process **(B)** MF, molecular function **(C)** CC, cellular component. **(D)** Top 10 significantly enriched pathways involved in sepsis and COVID-19 treatment with JHD by KEGG pathway enrichment analysis. Node color is displayed in a gradient from red to blue in descending order of the P-value.

The top 20 GO terms (according to *P*-value ranking) annotated to the BP, CC, and MF functional categories and the screened 10 significantly enriched pathways were visualized with bubble plots (Fig 3). The complete GO terms were presented in S3 Table.[Subxref6]

### Network construction of drug active ingredients and drug-disease targets

We intersected 456 drug targets, 4,271 sepsis disease targets, and 13,885 COVID-19 disease targets and obtained a total of 54 targets, suggesting that JHD might have the therapeutic effect by modulating the 54 target genes. In order to explore the action mechanism underlying JHD therapeutic effects, we inputted the information on these 54 targets into Cytoscape software and constructed a drug active ingredient-target network (S2 Fig). In the network, there were 657 nodes and 1,948 edges, and 44 drug active ingredients corresponded to 1,904 intersection targets. Topological analysis revealed high node degree values, indicating a high association between drug active ingredients and targets.

### Top 5 tissues or cells with highest expressions of key target genes are identified

A total of 37 genes were found to be significantly enriched in 10 key pathways, and based on these 37 genes, a network was constructed for topology analysis. Topology analysis revealed connectivity of nodes. Six key targets with degree values above the median degree were screened (Table 2). We found that these screened 6 key targets were overlapped with the intersection targets from the intersection of drug (JHD) targets and disease targets (sepsis and COVID-19). This finding might reveal the mechanism by which JHD treated different diseases, namely, JHD might regulate the same target genes which modulated different signaling pathways related to various diseases, thus achieving the treatments of different diseases.

**Table 2:** Six key target genes involved in sepsis and COVID-19 treatments with JHD.

Gene Symbol	Uniprot ID	Target protein name
*AKT1*	P31749	RAC-alpha serine/threonine-protein Kinase
*BCL2*	P10415	Apoptosis regulator Bcl-2
*ICAM1*	P05362	Intercellular Adhesion Molecule 1
*TLR4*	P35565	Toll-like Receptor 4
*MMP9*	P14780	Matrix Metalloproteinase-9
*HIF1A*	Q16665	Hypoxia-inducible Factor 1-alpha

**Fig 4:**
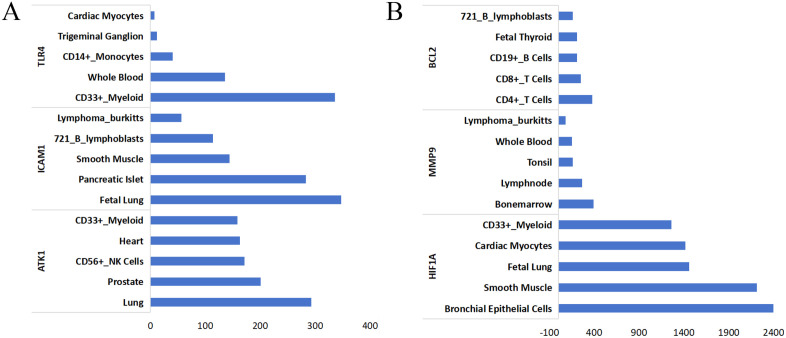
The tissue/cell distribution analysis. **(A-B)** Bar chart of tissue/cell distribution of six key target genes involved in in sepsis and COVID-19 treatments with JHD.

The bar chart of tissue/cell distribution of 6 key target genes was plotted according to the connectivity degree of node proteins (Fig 4). As shown in this distribution map, the top 4 tissues or cells with highest target gene expressions were CD33 + Myeloid, smooth muscle, fetal lung, and bronchial epithelial cells, respectively, which might play an important role in the treatment of both sepsis and COVID-19. Their discovery provides some guidance for the clinical target treatment of these two diseases. Target-tissue/cell network of 6 key target genes regulated in sepsis and COVID-19 treatments with JHD are shown in S3 Fig.[Subxref3]

### Validation of 6 key target genes by transcriptomics analysis

**Fig 5:**
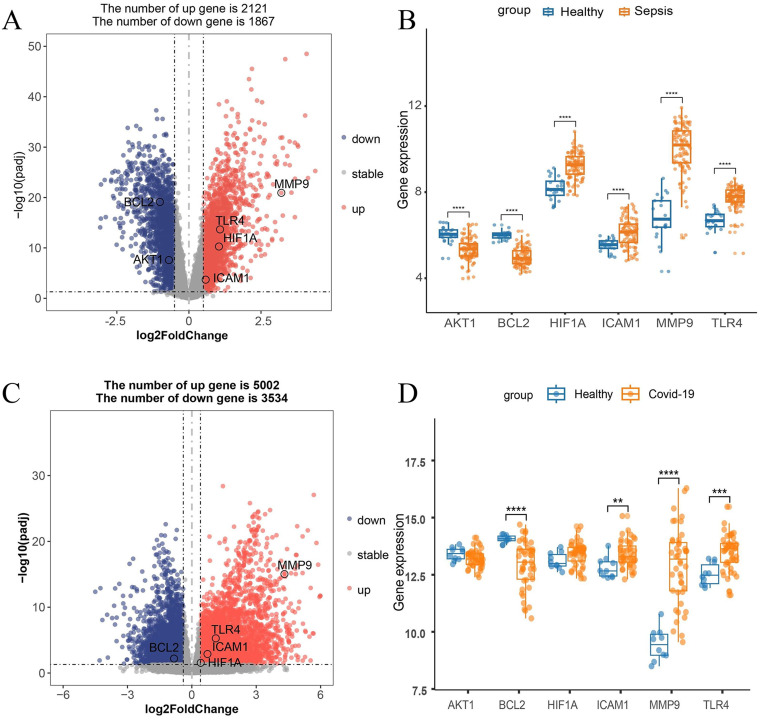
Validation of public transcriptome data. **(A)** Volcano plot of all differentially expressed genes (DEGs) in comparison of sepsis vs. healthy samples (GSE95233) (*P*-adj < 0.05 and |log_2_FC| > 0.5). **(B)** Box plot of differential expression of 6 key target genes in sepsis group vs. healthy group (****, *p *< 0.0001). **(C)** Volcano plot of all DEGs in COVID-19 group vs. Healthy group (GSE171110) (*P*-adj < 0.05 and |log_2_FC| > 0.4). **(D)** Box plot of differential expressions of 6 key target genes in in COVID-19 group vs. healthy group (**, *p* < 0.01; ***, *p* < 0.001; ****, *p* < 0.0001).

To further determine whether 6 key target genes identified in this study (*AKT1, MMP9, ICAM1, TLR4, BCL2,* and *HIF1A*) could be used as therapeutic targets for sepsis and COVID-19, we downloaded the gene expression profiles of sepsis and COVID-19 patients from the GEO database (GSE95233, GSE171110) (Fig 5). The gene differential expression analysis showed that compared with the healthy group, the sepsis patient group exhibited significantly lower expression levels of *AKT1* and *BCL2,* but significantly higher expression levels of *MMP9, ICAM1, TLR4,* and *HIF1A*. Compared with the healthy group, COVID-19 patient group displayed the significantly lower expression level of *BCL2*, but significantly higher expression levels of *MMP9, ICAM1,* and *TLR4*. The expression trends of *AKT1* and *HIF1A* in COVID-19 patients were consistent with those in sepsis patients. The results suggested that these six target genes might be potential therapeutic targets for sepsis and COVID-19.

## Discussion

The concept of “homotherapy for heteropathy” is a very peculiar and important therapeutic principle in TCM, the core idea of which is based on the holistic concept of Chinese medicine and the principle of targeting treatment based on pathogenesis of the disease, emphasizing that different diseases with the same pathogenesis or symptoms can be treated with the same treatment. In modern Chinese medicine practice, “homotherapy for heteropathy” has been widely used in clinical practice. With the in-depth exploration of the pharmacological effects of Chinese medicines in modern medicine, many Chinese medicines have been found to exhibit multiple regulatory functions, which provides scientific basis for “homotherapy for heteropathy”. In recent years, JHD has been proven to achieve desirable clinical efficacy in the treatment of both sepsis and COVID-19.

Sepsis and COVID-19 are clinically defined as epidemic febrile disorders according to TCM disease classification, with three predominant clinical manifestations of respiratory distress syndrome (PaO2/FiO2 < 200), multisystem organ failure, and inflammation. This pathophysiological complexity necessitates therapeutic strategies combining targeted pathogen eradication with host defense enhancement. Although sepsis and COVID-19 are different diseases, their pathogenesis is characterized by “toxin, stasis, and heat” in terms of traditional Chinese medicine pathology. JHD is composed of rhubarb, red vine, and dandelion, whose primary functions include clearing heat, removing toxin, activating blood circulation, clearing up bowels, and relieving swelling [32]. As a major component of JHD, rhubarb serves as the sovereign herb, known for its efficacy of purgation, detoxification, blood stasis resolution, and damp-heat clearing, making it a crucial medicine for heat clearing, and bowel movement. Red vine acting as the ministerial herb exhibits primary functions including clearing heat and detoxification, activating blood circulation, relieving pain, strengthening heat-clearing and toxin-removing effects of rhubarb. The adjunct herb dandelion is effective in clearing heat, detoxifying, relieving swelling, removing abscesses. In combination with other two herbs rhubarb and red vine, dandelion can enhance the efficacy of JHD in clearing heat and bowel movements. Intervention therapy on these common diseases reflects the characteristics of “homotherapy for heteropathy” by JHD.

In this study, based on our constructed drug-ingredient-target network, the main active ingredients of JHD were screened, primarily including tannins (from rhubarb), anthraquinones (from rhubarbol and rhubarbic acid), carboxylic acids, and flavonoids (from lignans and quercetin), which exhibited antibacterial, antioxidant, and anti-inflammatory effects [4]. Clinical and pharmacological studies have shown that anthraquinones have a broad-spectrum antibacterial effect, especially against bacteria *Staphylococcus aureus* and *Neisseria gonorrhoeae*, and anthraquinones have been reported to alleviate such symptoms as fever, pain, and swelling to a large extent [33,34]. Numerous studies have shown that rhubarbic acid can inhibit Toll-like receptor 2 (TLR-2) and peroxisome proliferator-activated receptor γ (PPARγ), and mediate the responses of inflammatory factors downstream the nuclear transcription factor-κB (NF-κB) pathway, including tumour necrosis factor-α (TNF-α), interleukin-1β (IL-1β), interleukin-6 (IL-6), intercellular adhesion molecule-1 (ICAM-1), and mesenchymal collagenase-1 (MMP-1) [35]. This study found that active ingredients in JHD such as rhubaric acid and lignans effectively alleviated inflammatory responses, which was consistent with previous reports that rhubarbic acid and lignans down-regulated expressions of pro-inflammatory factors such as IL-6, TNF-α, and IL-1β, inhibited the activation of MAPK/NF-κB signaling pathway, and relieved neutrophil infiltration and oxidative stress [36–38]. Additionally, dandelion flavonoids have been reported to ameliorate immune cell depletion by modulating CD4 + T cell activity and BCL2/BAX ratio [39].

In our study, 6 key target genes involved in sepsis and COVID-19 treatment with JHD were identified including *AKT1, MMP9, ICAM1, TLR4, BCL2* and *HIF1A* based on PPI network. Of them, *MMP9* has been reported to be primarily associated with inflammation and tissue remodeling in sepsis patients, while *MMP9* is closely related to the aggravation of acute respiratory distress syndrome (ARDS) and pulmonary fibrosis in COVID-19 patients, indicating that the *MMP9* had different functions in different diseases [40]. This study found that sepsis and COVID-19 shared similar pathological features, including excessive inflammatory response (cytokines storm), endothelial damage, immune dysregulation, and organ failure, implying that two diseases might be treated synergically by regulating common target genes.

*ICAM1,* as a cell adhesion molecule, is expressed in alveolar epithelial cells and vascular endothelial cells [41,42]. Consistently, this study found that in both sepsis and COVID-19, the inflammatory response resulted in *ICAM1* upregulation, further leading to acute lung injury (ALI) [43].

*TLR4* is a pattern recognition receptor in the immune system, and it is primarily responsible for recognizing molecules such as bacterial lipopolysaccharide (LPS) [44]. Our enrichment analysis identified *TLR4* as a hub molecule in the NF-κB signaling pathway, and this gene has been reported to be a core regulator of inflammation. In sepsis, the LPS-induced activation of the TLR4-NF-κB axis drives the cytokine storm. [45,46]. We speculated that in sepsis, this cytokines storm might trigger an autoimmune attack, thus causing acute respiratory distress syndrome and multiple organ failure, finally aggravating sepsis. Similarly, for COVID-19, *TLR4* triggers an excessive inflammatory response, exacerbating severe acute respiratory syndrome coronavirus 2 (SARS-CoV-2) spiking protein-mediated lung injury, thereby deteriorating COVID-19. Our gene differential expression analysis showed that compared with the healthy group, both sepsis and COVID-19 patient groups exhibited the significantly upregulated expression levels of *TLR4*, suggesting that synergistic treatment of these two diseases could be implemented by regulating *TLR4* target gene.

*ATK1*, a central kinase in the PI3K-AKT signaling pathway, can reduce neutrophil recruitment and prevent acute lung injury [47–49]. In this study, the tissue or cell distribution analysis of the 6 key target genes revealed that *ATK1* was significantly expressed in the lung. Our data also indicated that *ATK1* was a key treatment target for sepsis and COVID-19 in the protein interaction network. This is consistent with recent reports that *ATK1* is identified as a key treatment target for COVID-19. Within the PI3K-AKT pathway framework, *AKT1* enhances the anti-apoptotic function of *BCL2* gene by phosphorylating and inhibiting pro-apoptotic proteins, highlighting the synergistic role of these two genes in regulating the immune response to sepsis and COVID-19 [46,50]. This is supported by our findings that the expression pattern of *AKT1* was consistent with that of *BCL2* in both sepsis and COVID-19.

*HIF1A*, the central transcriptional regulator of the HIF-1 signaling pathway, not only mediates cellular responses to hypoxia but also plays a central role in regulating inflammatory responses [51,52]. Our gene differential expression analysis revealed that compared with the healthy group, COVID-19 and sepsis groups exhibited the significantly upregulated expression of this gene. Based on these findings, we speculated that *HIF1A* might trigger a high inflammatory response in both diseases. In addition, *HIF1A* has also been reported to promote SARS-CoV-2 infection and exacerbate the inflammatory response in COVID-19, and SARS-CoV-2-induced cytokine storms are regarded as a major pathological feature of COVID-19. Taken together, increasing evidence supports that *HIF1A* plays a pivotal role in regulating immune and inflammatory responses, and thus *HIF1A* gene can act as target to effectively control high inflammatory response [53].

In addition, our study utilized the BioGPS tool to analyze the tissue/cell distribution of 6 key target genes and found that these 6 target genes were primarily distributed in fetal lung and bronchial epithelial cells, and their distribution in tissue was highly overlapped with lesion location of sepsis and COVID-19. This study identified the tissue/cell distribution of 6 key target genes playing critical roles in the pathophysiology of sepsis and COVID-19. In sepsis and COVID-19, excessive activation of CD33 + myeloid cells triggered abnormal inflammation responses, thus resulting in immunosuppression, which represented typical pathogenesis of these two diseases. The sepsis-induced inflammatory response has been reported to increase vascular permeability and vascular tone dysregulation [54]. Severe pulmonary lesions in COVID-19 are often accompanied by pulmonary vascular alterations which are closely associated with dysfunction of smooth muscle cells [55]. Both sepsis and COVID-19 can lead to detachment of bronchial epithelial cells and their damage, and this respiratory epithelial cell damage is a hallmark of acute lung injury (ALI) and acute respiratory distress syndrome (ARDS) [42]. Upon viral invasion, bronchial epithelial cells, as the major target cells for SARS-CoV-2 infection, undergo damage and cytokine release, thereby triggering inflammatory responses [56]. Fetal lung cells are frequently employed to mimic the airway microenvironment *in vitro* and to investigate intercellular interactions, and the constructed airway models are of great value for the treatment of sepsis and COVID-19 [57]. This finding further demonstrated that JHD could achieve the “homotherapy for heteropathy” based on the shared pathogenesis mechanism of different diseases.

In conclusion, this study found that JHD might achieve “homotherapy for heteropathy” for sepsis and COVID-19 through multi-ingredient, multi-target, and multi-pathway therapies, further confirming that these two diseases were interrelated in therapeutic mechanism. Our findings provide valuable references for subsequent animal experimental verification and clinical application of JHD to sepsis and COVID-19 as well as its potential expanded application to other related disease.

Although our integrated analysis provides molecular-level evidence for achieving “homotherapy for heteropathy” by JHD, there are several limitations. Firstly, the integration of datasets may generate redundant information and lead to potential biases, so we implemented deduplication steps during data processing (merging gene symbols and removing identical targets). Secondly, our findings mainly rely on database predictions and computational simulations, and thus subsequent experimental validation is needed. Lastly, the network pharmacology method possibly oversimplifies biological complexity of JHD, thereby resulting in the overestimation of drug-likeness and neglection of off-target effects. Therefore, animal and clinical experiments should be conducted to validate the proposed mechanisms and therapeutic efficacy of JHD against sepsis and COVID-19 in the future.

## Supporting information

S1 Fig Protein–protein interaction (PPI) network. Protein-protein interaction (PPI) Network Diagram of 54 intersection (drug-disease) Targets from STRING database.
(TIF)

S2 Fig The drug-target interaction pharmacology network. Drug active ingredient-target network. The central diamonds represent intersection targets (1,904), and 3 circles indicate drug active ingredients of 3 traditional Chinese medicine components (rhubarb, red vine, and dandelion) in JHD. Node shape size and node color depth were positively correlated with the association degree between drug active ingredients and targets.
(TIF)

S3 Fig Target-tissue/cell network. Target-tissue/cell network of 6 key target genes regulated in sepsis and COVID-19 treatments with JHD.
(TIF)

S1 Table Abbreviations list. (XLSX)

S2 Table Summary of GEO datasets for patients with sepsis or COVID-19. (XLSX)

S3 Table Summary of GO and KEGG pathway enrichment terms. (XLSX)
