# Supplementary figures and images for: Analysis of molecular mechanism of Chinese medicine Jinhong decoction (JHD) in synergistically treating sepsis and COVID-19 based on network pharmacology
Source: PLoS One. 2025 Dec 18;20(12):e0339457. doi: 10.1371/journal.pone.0339457 (PMC12714189; doi:10.1371/journal.pone.0339457)

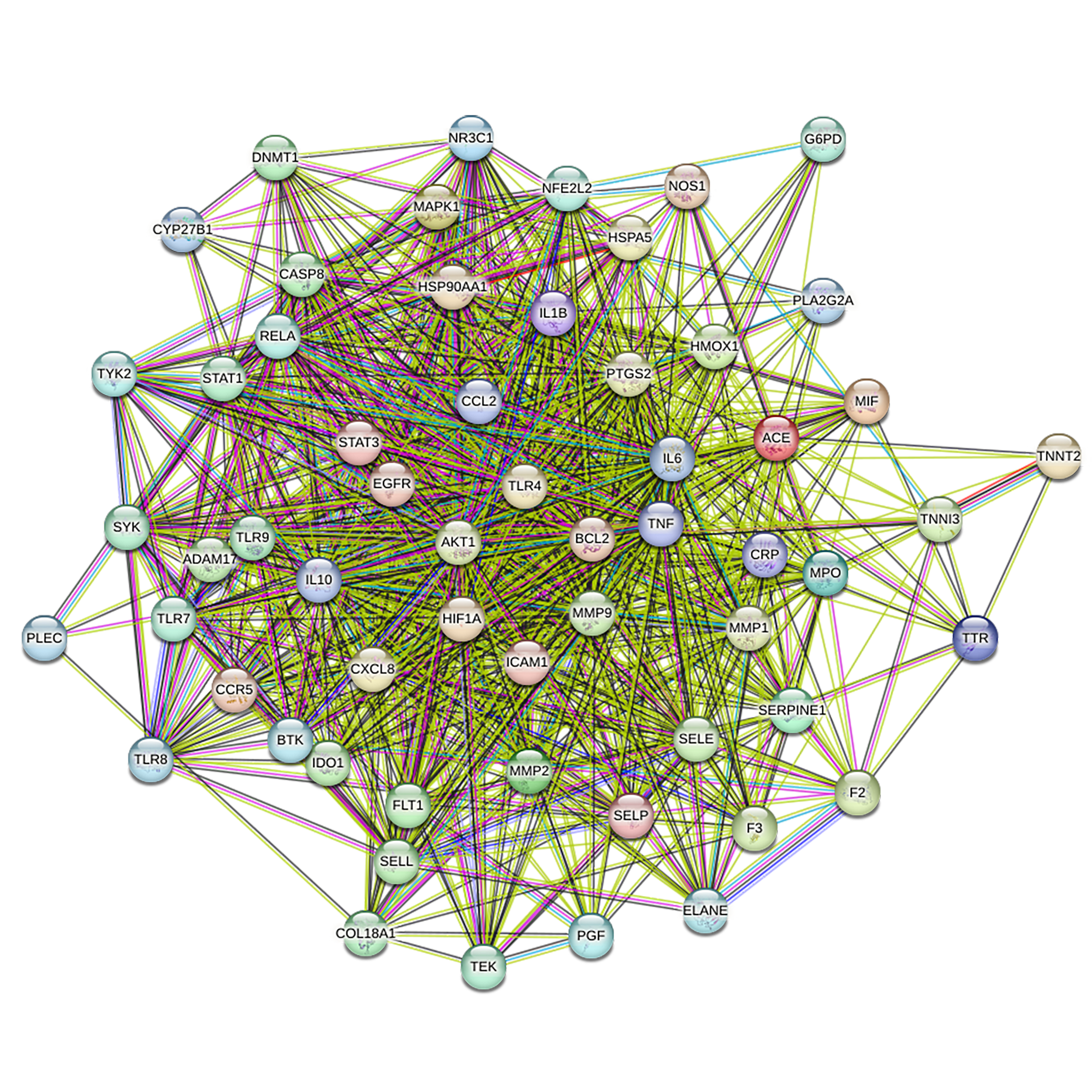

Supplement: S1 Fig — Protein-protein interaction (PPI) Network Diagram of 54 intersection (drug-disease) Targets from STRING database. (TIF) [file pone.0339457.s001.tif]

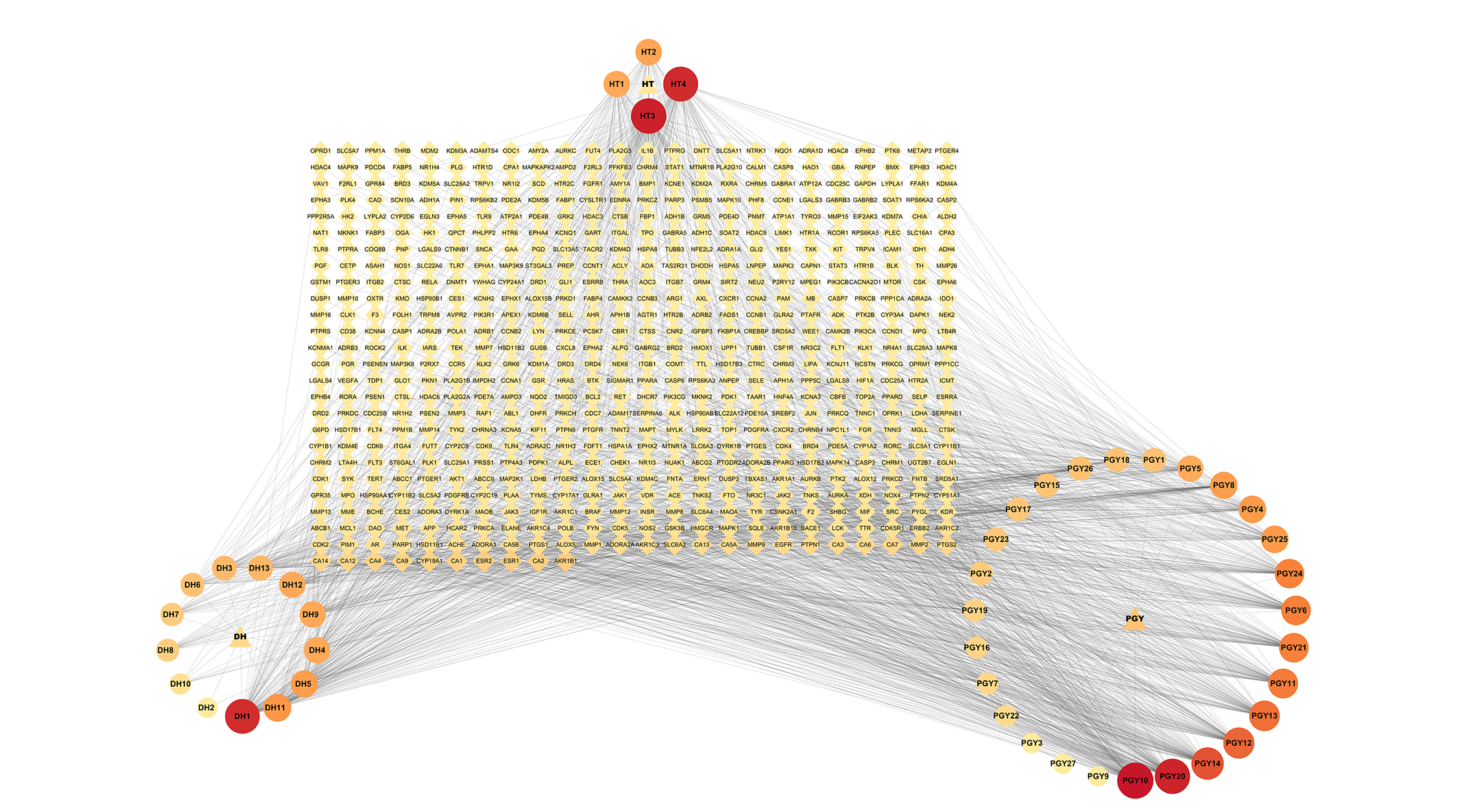

Supplement: S2 Fig — Drug active ingredient-target network. The central diamonds represent intersection targets (1,904), and 3 circles indicate drug active ingredients of 3 traditional Chinese medicine components (rhubarb, red vine, and dandelion) in JHD. Node shape size and node color depth were positively correlated with the association degree between drug active ingredients and targets. (TIF) [file pone.0339457.s002.tif]

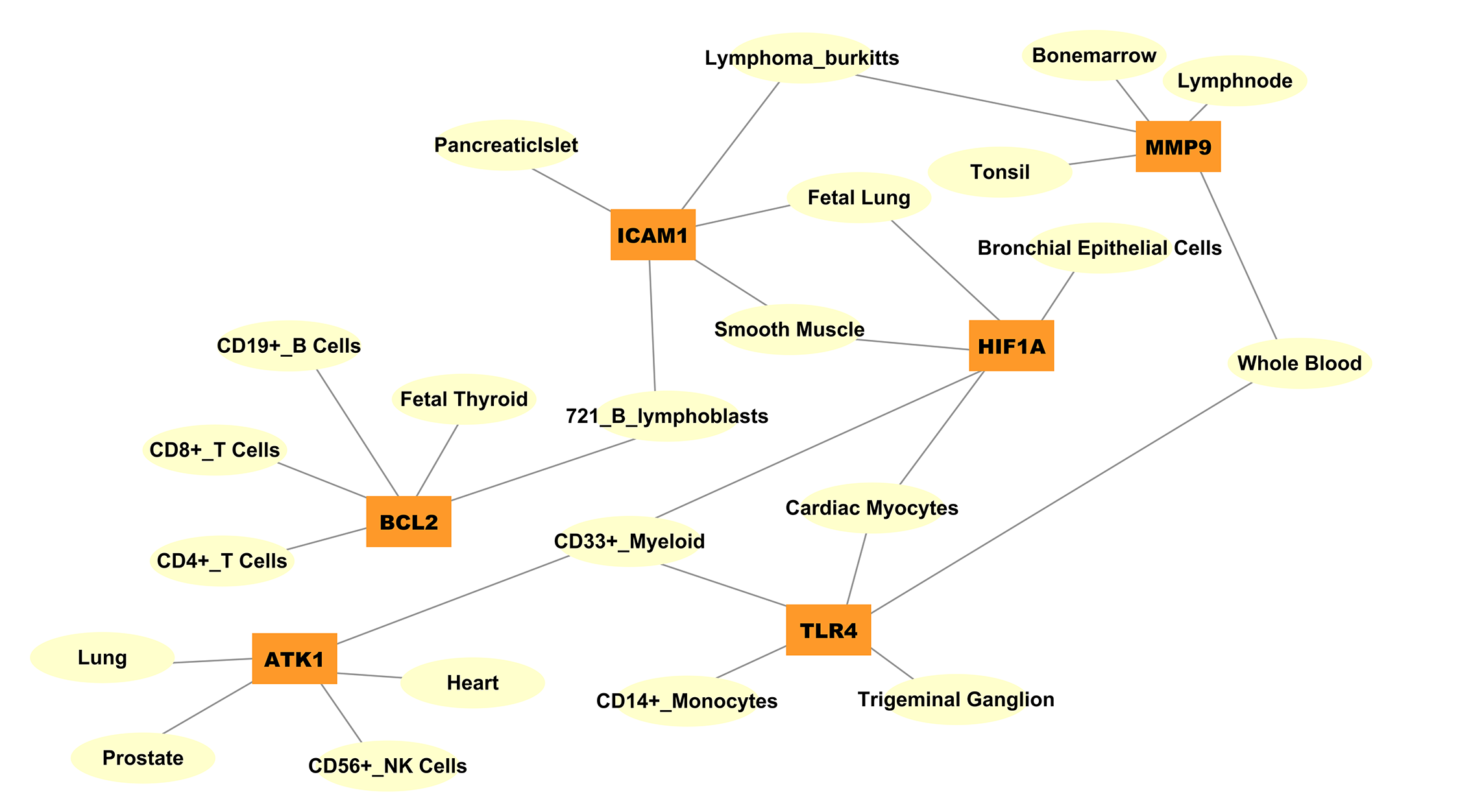

Supplement: S3 Fig — Target-tissue/cell network of 6 key target genes regulated in sepsis and COVID-19 treatments with JHD. (TIF) [file pone.0339457.s003.tif]
